# Coating of manganese functional polyetheretherketone implants for osseous interface integration

**DOI:** 10.3389/fbioe.2023.1182187

**Published:** 2023-05-03

**Authors:** Xin Yang, Shouliang Xiong, Jing Zhou, Yinchang Zhang, Huazheng He, Pingbo Chen, Congming Li, Qiang Wang, Zhiqiang Shao, Lei Wang

**Affiliations:** ^1^ Department of Orthopedics, The First Affiliated Hospital of Wannan Medical College, Wuhu, Anhui, China; ^2^ Orthopedics and Sports Medicine Center, The Affiliated Suzhou Hospital of Nanjing Medical University, Suzhou, China

**Keywords:** PEEK, manganese, polydopamine, biocompatibility, osseointegration

## Abstract

Polyetheretherketone (PEEK) has been used extensively in biomedical engineering and it is highly desirable for PEEK implant to possess the ability to promote cell growth and significant osteogenic properties and consequently stimulate bone regeneration. In this study, a manganese modified PEEK implant (PEEK-PDA-Mn) was fabricated via polydopamine chemical treatment. The results showed that manganese was successfully immobilized on PEEK surface, and the surface roughness and hydrophilicity significantly improved after surface modification. Cell experiments *in vitro* demonstrated that the PEEK-PDA-Mn possesses superior cytocompatibility in cell adhesion and spread. Moreover, the osteogenic properties of PEEK-PDA-Mn were proved by the increased expression of osteogenic genes, alkaline phosphatase (ALP), and mineralization *in vitro*. Further rat femoral condyle defect model was utilized to assess bone formation ability of different PEEK implants *in vivo*. The results revealed that the PEEK-PDA-Mn group promoted bone tissue regeneration in defect area. Taken together, the simple immersing method can modify the surface of PEEK, giving outstanding biocompatibility and enhanced bone tissue regeneration ability to the modified PEEK, which could be applied as an orthopedic implant in clinical.

## 1 Introduction

Due to trauma, osteoporotic fractures or tumor resections in orthopedic field result in a large number of patients needing bone grafts and surgical repair every year (Wang et al., 2018; Geng et al., 2022). Existing bone repair materials include autogenous bone, allogeneic bone and artificial bone graft materials. Artificial bone grafting materials have great promise in medical field, but also face great challenges (Li et al., 2018). Artificial bone grafting materials include metal, ceramic and polymer materials. Titanium and titanium alloy are commonly employed metals as bone graft materials due to their superior biocompatibility, corrosion resistance as well as flexibility. However, stress shielding phenomenon, wear, corrosion and eventually implant migration of titanium and titanium alloy restrict the development of clinical application of metal materials (Geng et al., 2021). Ceramic biomaterials are expected to replace autogenous bone grafts. However, the excessive brittleness and low strength of bioactive ceramic seriously affect their performance as bone repair material (Gao et al., 2017). The functions of elastic modulus, bioactivity and osteogenic induction are the key to evaluating the properties of biomaterials. The good elastic modulus makes the biomaterial and bone tissue better match and avoids the stress shielding effect (de Ruiter et al., 2021). In addition, good biological activity is conducive to cell adhesion and proliferation on biomaterials surface (Sang et al., 2022). The bone-inducing properties of biomaterials stimulate the ability of osteogenesis and stimulate osteoblasts to form new bone tissue (Qi et al., 2021).

At present, Polyetheretherketone (PEEK) is an excellent candidate for repairing bone defects due to the superiority of excellent biocompatibility, elastic modulus equivalent to natural bone, chemical stability, X-ray permeability, and ease-of-processing (Panayotov et al., 2016; Chen et al., 2022). PEEK has been commonly applied in trauma and orthopedics, but its weak osteogenic ability is the main factor affecting its long-term stabilization and repair effect (Zhang et al., 2021). Because natural PEEK is a biologically inert material, its surface needs to be modified to promote its integration with adjacent bone tissue. Enhancing the osteogenic activities of PEEK implants with surface modification is an efficient method (Buck et al., 2020). Various physical or chemical modification techniques could enhance the osteogenic properties of PEEK by dispersing active metal ions on the surface of the material to meet clinical use requirements. Sun et al. (2021) reported that strontium modified 3D porous sulfonated PEEK via hydrothermal treatment can enhance the adhesion, alkaline phosphatase activity, secretory of collagen and extracellular mineralization of osteoblasts *in vitro*. Deng et al. (2018) proved that the preparation of double Ag/Zn ions modified sulfonated PEEK by layer self-assembly strategy has excellent antibacterial ability and biocompatibility, which effectively suppressed the proliferation of Gram-positive and Gram-negative bacteria, and enhanced the biocompatibility of MG-63 cells and accelerates bone differentiation and maturation.

Manganese (Mn) has drawn increasing interest in a range of biomedical applications given its promising biologically active properties. As an essential trace element, manganese is related to maintaining bone structure and regulating bone metabolism (Li et al., 2021a). Studies have found that the absence of Mn element during bone formation disrupts the dynamic equilibrium between osteoclasts and osteoblasts and the content of chondroitin sulfate is reduced, which leads to osteoporosis (Park et al., 2011). Human osteoblast integrins have been shown to be activated by Mn, which has dose-dependent effects on enhancing cell adhesion, proliferation and diffusion (Cassari et al., 2022). Zhao et al. (2020) prepared a manganese doped TiO_2_ microporous coating with good bone integration effect, which has been proven through animal experiments. Breno et al. reported that manganese-modified bioactive glass has a broad variety of potential uses for promoting bone tissue repair (Barrioni et al., 2019). Recently, Li et al. (2021b) reported that manganese containing bioceramic materials having the capacity to promote osteoblast differentiation, scavenge reactive oxygen species (ROS) and inhibit bone absorption might be the ideal choice for the therapy of osteoporotic bone defect. These works suggest that adding manganese to implants is an efficient strategy to impart osteogenic activity to implants.

Several physical and chemical methods have been reported to incorporate manganese into various surfaces of biomaterials to promote osteogenic activity, such as micro-arc oxidation (MAO) (Zhao et al., 2020), sol-gel method (Barrioni et al., 2019), coprecipitation method (Li et al., 2021b), plasma immersion ion implantation and deposition (PIII&D) technique (Yu et al., 2017). However, most of these methods require particular devices, complex operating procedures and complicated reaction conditions, thus limiting their practical application. Therefore, we wondered whether manganese ions could be introduced into the PEEK surface in a simple and environmentally friendly way to prepare implant materials with the biological effect of promoting osteogenesis. Polydopamine (PDA) is a kind of natural melanin with a chemical structure similar to the mucin secreted by marine mussels, in which the catechol group has strong adhesion properties and polydopamine coatings can be bonded via covalent and/or non-covalent bonding to the surface of organic or inorganic materials with different properties (Lee et al., 2009; Wang et al., 2021). Currently, the chemical modification technology of polydopamine is widely used to modify various biomaterials and provide an active platform for the secondary modification of biomaterials (Lu et al., 2018; Barros et al., 2021). Previous studies have shown that polydopamine coatings can chelate active metal ions (including Mg, Cu, and Zn) and be immobilized on the surface of various biomaterials (Wang et al., 2017; Wang et al., 2019; Wei et al., 2023).

**FIGURE 1:**
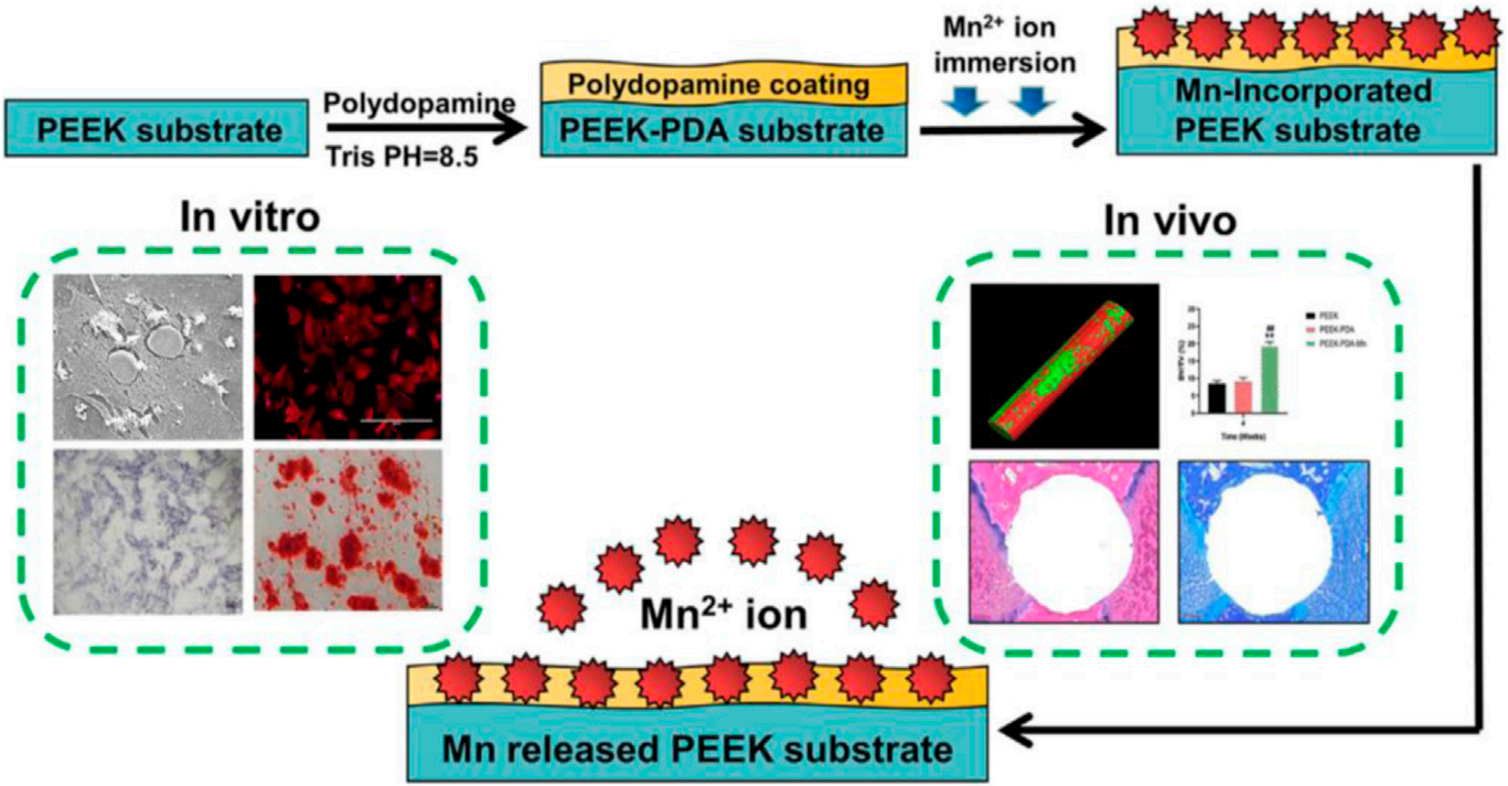
Schematic diagram of the process used for preparation and evaluation of the PEEK-PDA-Mn materials.

In this study, the PEEK implant with hydrophilicity and biological activity was enhanced by the chemical surface modification of PDA and further chelation of manganese ions (Figure 1). Therefore, three kinds of samples, pure PEEK implant (PEEK), PEEK implant with PDA coating on surface (PEEK-PDA) and PEEK implant doped with manganese ions (PEEK-PDA-Mn) were prepared. *In vitro* study, various PEEKs were co-cultured with MC3T3-E1 cells to evaluate the cytocompatibility, cell adhesion and osteogenesis of various PEEKs. *In vivo* study, PEEK-PDA-Mn implant was implanted into the rat femoral bone defect model and the bone regeneration capacity of various PEEKs was observed by Micro-CT and histological analysis.

## 2 Materials and methods

### 2.1 Fabricating various PEEKs

PEEK discs with diameters of 5.8, 13, and 21.4 mm were used for *in vitro* cytological experiments. In addition, PEEK discs of size Φ 1.2 mm × 10 mm were applied for *in vivo* animal studies. Each type of PEEK was purged using acetone, ethanol and deionized water in turn with ultrasonic treatment. Finally, all PEEK were stored in a dryer after drying at ambient temperature.

Firstly, dopamine (Sigma, United States) was added into Tris-HCl solution (10 mM) at pH 8.5 to prepare dopamine solution with a concentration of 2 mg mL^−1^. The PEEK was subsequently soaked in dopamine solution over 12 h to prepare PDA-coated PEEK (PEEK-PDA), and then cleaned with deionized water under ultrasonication. Afterwards, the PEEK-PDA was dried at room temperature and stored for further experiments. PEEK-PDA was treated with MnCl_2_ solution (1.25 mg/mL, Yuanye, China) for 1 h to obtain Mn decorated PEEK-PDA (PEEK-PDA-Mn). At last, all samples were cleaned with deionized water, dried at room temperature and autoclaved for the following experiments.

### 2.2 Characterization

The morphology and roughness of PEEK, PEEK-PDA and PEEK-PDA-Mn surface were studied using scanning electron microscope (SEM, S-3400, Hitachi, Japan) and atomic force microscopy (AFM, XE-100, Park Systems, United States). The chemical elements of various PEEKs were characterized using energy dispersive X-ray spectroscopy (EDS, QX200, Bruker, Germany). The hydrophilic property of various PEEKs was measured by contact analysis system (DSA 25S, Kruss GmbH, Germany); Three parallels were investigated for each group.

### 2.3 Mn ion release

Phosphate buffered solution (10 mL; PBS, Hyclone, United States) was added to the PEEK-PDA-Mn sample and incubated at 37°C for 1, 3, 5, 7,14, and 21 days without stirring. These periods were selected based on the *in vitro* biocompatibility tests. At each time point, inductively coupled plasma atomic emission spectrometry (ICP-AES, Leeman, United States) was used to quantify the Mn^2+^ release. Three parallels were investigated for each test.

### 2.4 Cell culture

MC3T3-E1 cells (CRL-2594, Chinese Academy of Science, China) were utilized to study the biocompatibility of various PEEKs. MC3T3-E1 cells were cultivated in α-MEM (Gibco, United States) supplied with 10 vt% FBS and 1 vt% penicillin/streptomycin in a humid incubator at 37°C with 95% relative humidity and 5% CO_2_ partial pressure.

### 2.5 Cell morphology observation

MC3T3-E1 cells of 1 × 10^4^ cells/well were inoculated on the surface of the various PEEKs in a 96-well plate for 24 h. The cells were then washed by PBS (Hyclone, United States) and incubated in glutaraldehyde (2.5%, Sigma, United States) in a humid incubator at 37°C for 1 h. The cells were cleaned with PBS 3 times, then sequentially dehydrated by ethanol at graded concentrations (30%, 50%, 70%, 90%, and 100%). Each concentration lasted for 30 min. After being treated with a critical point dryer (CPD030, Leica, Germany) and iron sputtering (SC7620, Quorum Technologies, UK), the cell morphology on various PEEKs was examined by SEM.

### 2.6 Cell cytoskeletal observation

MC3T3-E1 cells of 2 × 10^4^ cells/well were inoculated on various PEEKs surfaces in 24-well plate for 24 h. Then, MC3T3-E1 cells were rinsed with PBS, following 20-min fixation in 4% paraformaldehyde (PFA, Sigma, MO) and PBS washing. After that, 0.2% Triton X-100 (Sigma, United States) was applied to MC3T3-E1 cells for 25 min and blocked with 0.1% bovine serum albumin (BSA, Sigma, United States) for photoprotection at ambient temperature. Finally, the cytoskeleton was stained with 4′6-diamidino-2-phenylindole (DAPI, Beyotime, China) and rhodamine-phalloidin (Molecular Probes, OR), and the cytoskeleton was imaged with a fluorescence microscope (AMG, United States).

### 2.7 Cell viability assays

CCK-8 (CCK-8, Dojindo Laboratories, Japan) was utilized to evaluate the cell viability on different PEEK surfaces. In 96-well plates, the cells of 1 × 10^4^ cells/well were pipetted on PEEK surface. Following incubation for 1, 4, and 7 days, the culture medium’s optical density (OD) was quantified with the CCK-8 method utilizing a spectrophotometric microplate reader (Bio Rad 680, United States), as per manufacturer’s instructions. The relative growth rate of the cells was quantitatively determined by formulation.

$$Relative\left(RGR\right)=\frac{Optical\;density{\left(OD\right)}_{samples}}{Optical\;density{\left(OD\right)}_{blank}}\times 100\%$$

Where OD_*blank*_ is the OD of blank control group (α-MEM medium) and OD_*sample*_ is the OD of the samples.

### 2.8 Osteogenic genes expression

MC3T3-E1 cells of 1 × 10^5^ cells/well were co-cultured on various PEEKs in 6-well plates. After incubation for 7 and 14 days, cells on various PEEKs were lysed with TRIzol reagent (Invitrogen, United States) to obtain total ribonucleic acid (RNA), and the amount of the collected RNA was evaluated with UV/VIS. Complementary deoxyribonucleic acid (DNA) is prepared from 1 mg of collected RNA by the IScript™cDNA Synthesis Kit. Primers for alkaline phosphatase (ALP), osteopontin (OPN), dwarf related transcription factor 2 (Runx2), collagen-I (Col-Ⅰ) and osteocalcin (OCN) genes are shown in Table 1. ABI Prism 7500 Thermal Cycles and SsoAdvanced SYBR Green Supermix reagent (BIORAD Technologies) were applied to evaluate the osteogenic messenger RNA (mRNA) expression level. Using the ΔΔCt method, relative expression was calculated and normalized to *β*-Actin (housekeeping gene) expression.

**TABLE 1:** The primer sequences.

Gene	Forward (5′-3′)	Reverse (5′-3′)
ALP Runx2	TAT​GTC​TGG​AAC​CGC​ACT​GAA​C ATC​CAG​CCA​CCT​TCA​CTT​ACA​CC	CGG​GAC​CAT​TGG​GAA​CTG​ATA​GG ACT​AGC​AAG​AAG​AAG​CCT​TTG​G
OPN	GCG​GTT​CAC​TTT​GAG​GAC​AC	TAT​GAG​GCG​GGG​ATA​GTC​TTT
ColⅠ	CAG​GCT​GGT​GTG​ATG​GGA​TT	CCA​AGG​TCT​CCA​GGA​ACA​CC
OCN	AAC​GGT​GGT​GCC​ATA​GAT​GC	AGG​ACC​CTC​TCT​CTG​CTC​AC
β-actin	CTC​ATG​CCA​TCC​TGC​GTC​TG	GGC​AGT​GGC​CAT​CTC​TTG​CT

### 2.9 ALP and alizarin red staining

MC3T3-E1 cells of 1 × 10^4^ cells/well were cultured on PEEK surface for 7 and 14 days, respectively. Following fixation by 4% paraformaldehyde, the staining of the samples was performed using ALP (Beyotime, China) and alizarin red staining kits (Cyagen) according to the instructions. The stained samples were then examined using a microscope. (DMi8, Leica, Germany).

### 2.10 Evaluating PEEK *in vivo*

The animal experiment was carried out following the NIH guidelines, and permitted by the Institutional Animal Care and Use Committee of Wannan Medical College (Anhui, China). There were twelve 300–350 g male SD rats assigned into three groups at random. Prior to surgery, rats were treated with anesthesia and disinfection. The lateral femoral condyle of left knee was exposed through a progressively slit. Then, the longitudinal bone defect of the femoral condyle with 1.2 mm diameter and 10 mm height was created on left side using a dental drill. Finally, the autoclaved various PEEK was implanted into the defect area, followed by suturing with 4-0 nylon. After 4 weeks, all rats were euthanized by inhaling CO_2_ and samples were harvested for micro-computed tomography (Micro-CT) scanning and histological analysis.

### 2.11 Micro-CT analysis

Micro-CT (SkyScan 1176, SkyScan, Aartselaar, Belgium) was utilized to observe the regenerated bone tissue around various PEEKs under the fixed parameter conditions (385 mA, 65 kV and AI filter of 1 mm). High-resolution 3D image was provided through the manufacturer’s software (SkyScan CTVOX 2.1).

### 2.12 Histological analysis

After implantation for 4 weeks, all collected femur specimens were fixed with 4% paraformaldehyde. Bone tissue was then decalcified using 10% EDTA solution for 28 days. Then, the samples were gently separated from the femur, and the femur was progressively dehydrated by graded ethanol before being covered in paraffin wax. The femur was machined into 5 μm thick cross-section. Finally, the section was treated with hematoxylin-eosin (H&E) and Masson staining. The stained section was viewed and captured by a light microscope (Nikon 80i, Nikon, NY).

### 2.13 Statistical analysis

All data were analyzed by SPSS 20 statistical software (version 20.0) and significant differences were analyzed using ANOVA with Tukey’s post-test. The data were expressed as mean ± standard deviation (SD) for *n* = 3 independent experiments *in vitro* and *n* = 4 independent experiments *in vivo*. Within each figure panel, *p* values on graphs are indicated as *P 0.05 (significant) and **P 0.01 (moderately significant), respectively.

## 3 Results

### 3.1 Material surface characterization

**FIGURE 2:**
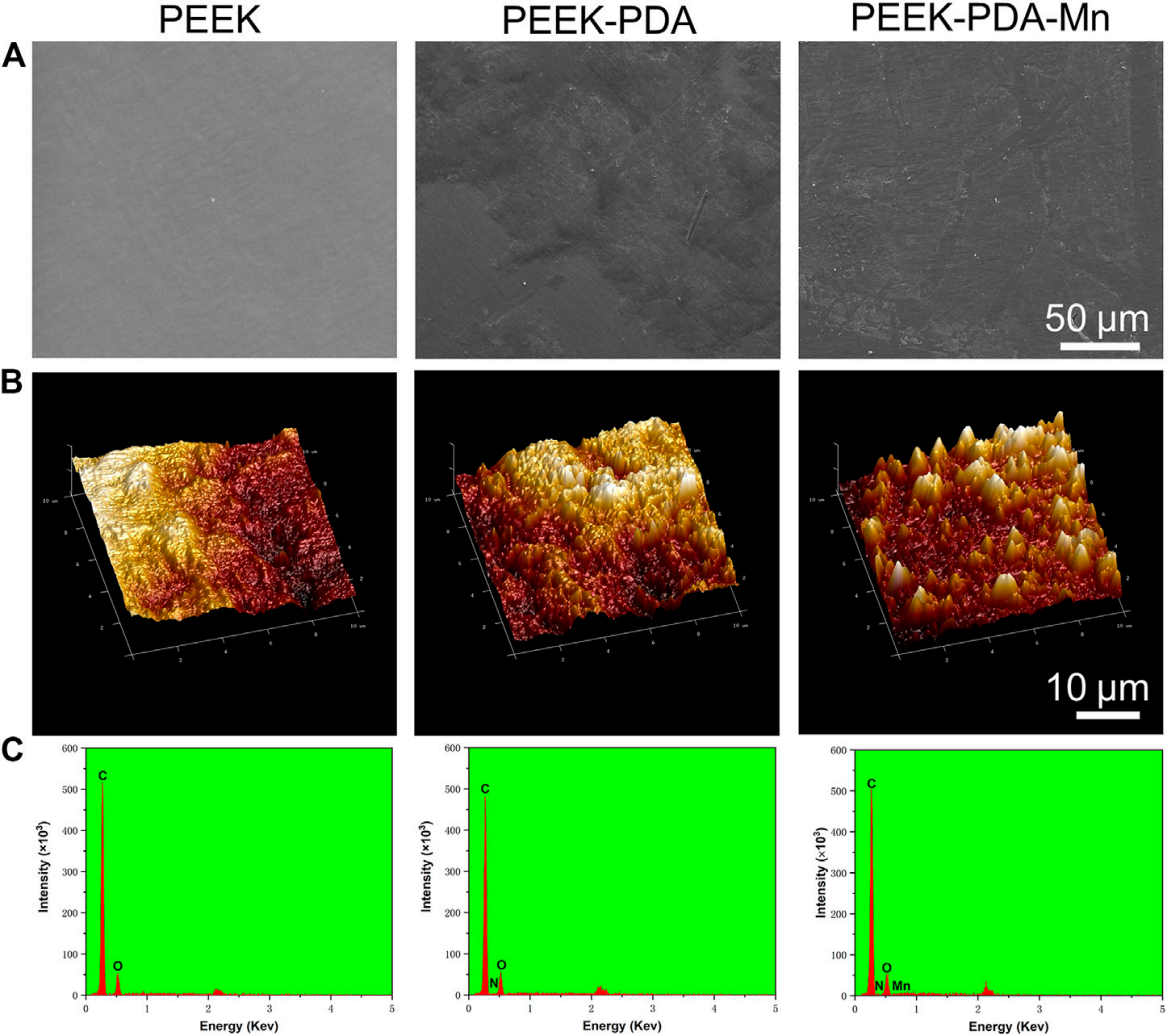
**(A)** SEM images of PEEK, PEEK-PDA and PEEK-PDA-Mn. Scale bars, 50 μm. **(B)** 3D topographical AFM images of PEEK, PEEK-PDA and PEEK-PDA-Mn. Scale bars, 10 μm. **(C)** EDS spectrum of PEEK, PEEK-PDA and PEEK-PDA-Mn.

**FIGURE 3:**
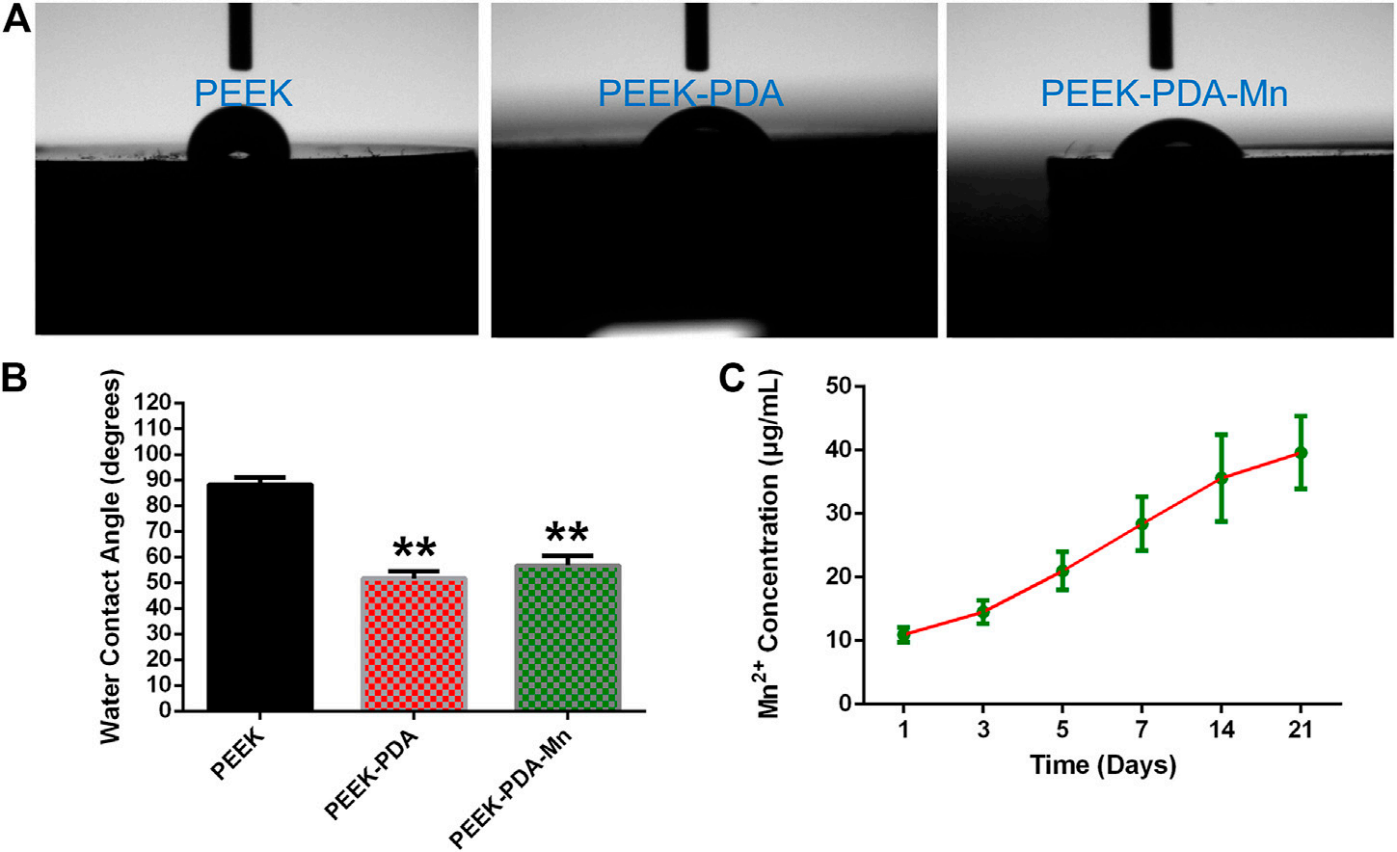
**(A)** Representative pictures of water droplets on different PEEKs. **(B)** Water contact angles of PEEK, PEEK-PDA, and PEEK-PDA-Mn substrates. **p < 0.01 vs. PEEK. **(C)** Cumulative released concentrations of Mn^2+^ from PEEK-PDA-Mn samples for 1, 3, 5, 7, 14, and 21 days.

The morphology of various PEEKs surfaces was examined through SEM (Figure 2A). The pure PEEK displayed smooth surface morphology. In contrast, PEEK-PDA-Mn and PEEK-PDA displayed rougher surface, suggesting that the surface morphology of PEEK was changed through the surface modification of polydopamine and manganese ions. AFM further confirmed these morphological differences (Figure 2B). The roughness (Rq) of pure PEEK significantly increased after polydopamine treatment from 21.8 to 26.3 nm. After incorporation of Mn, the Rq of PEEK-PDA-Mn increased to 34.7 nm. The EDS results demonstrated the successful incorporation of Mn ions onto PEEK-PDA-Mn surface, as evidenced by the existence of Mn elements in comparison to PEEK-PDA and PEEK (Figure 2C). The successful coating of PDA was ascribed to the EDS results that N was observed in the other two groups except PEEK group. In a further, PEEK-PDA-Mn group has 5.54% Mn^2+^ (w/w). The C signal detected in all groups was primarily ascribed to PEEK and PDA. The surface hydrophilicity of PEEK was changed by surface chemical modification (Figures 3A, B). Compared to the hydrophilic surface of PEEK (88.2° ± 1.66°), the contact angles of the PEEK-PDA (51.63° ± 1.64°) and PEEK-PDA-Mn (56.73° ± 2.13°) decreased significantly, indicating that the PDA coating considerably increased the hydrophilicity of PEEK surface.

### 3.2 Mn ions release

To investigate the release behavior of Mn^2+^, the amount of released Mn^2+^ from PEEK-PDA-Mn was evaluated using ICP-AES measurement (Figure 3C). In the test of the first 24 h, PEEK-PDA-Mn samples released 10.92 ± 0.66 μg/mL of Mn^2+^. After release for 7 days, about 28.39 ± 2.45 μg/mL of Mn^2+^ released from the samples. A relatively fast Mn^2+^ release was observed in the first 7 days and subsequently the release rate progressively decreased. By the end of the ion release measurement, the final concentration of Mn^2+^ reached 39.61 ± 3.31 μg/mL after being immersed for 21 days.

### 3.3 Cell morphology

**FIGURE 4:**
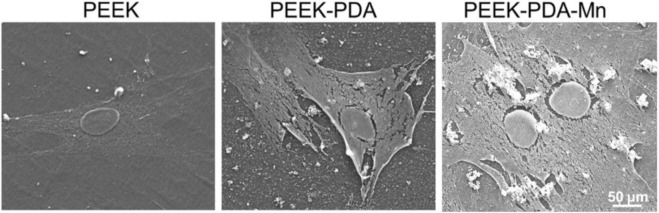
Cell morphology of MC3T3-E1 cells cultured on PEEK, PEEK-PDA, and PEEK-PDA-Mn substrates for 24 h, as observed using SEM. Scale bars, 50 μm.

After 24 h of culture, MC3T3-E1 cells on various PEEKs surface showed varied morphology (Figure 4). It is observed that the cells on PEEK surface exhibited a relatively spherical shape. However, the cells on PEEK-PDA-Mn and PEEK- PDA surface showed a flatter, spread and irregular shape on the material surface, indicating that the PDA coating on PEEK surface improved its biocompatibility.

**FIGURE 5:**
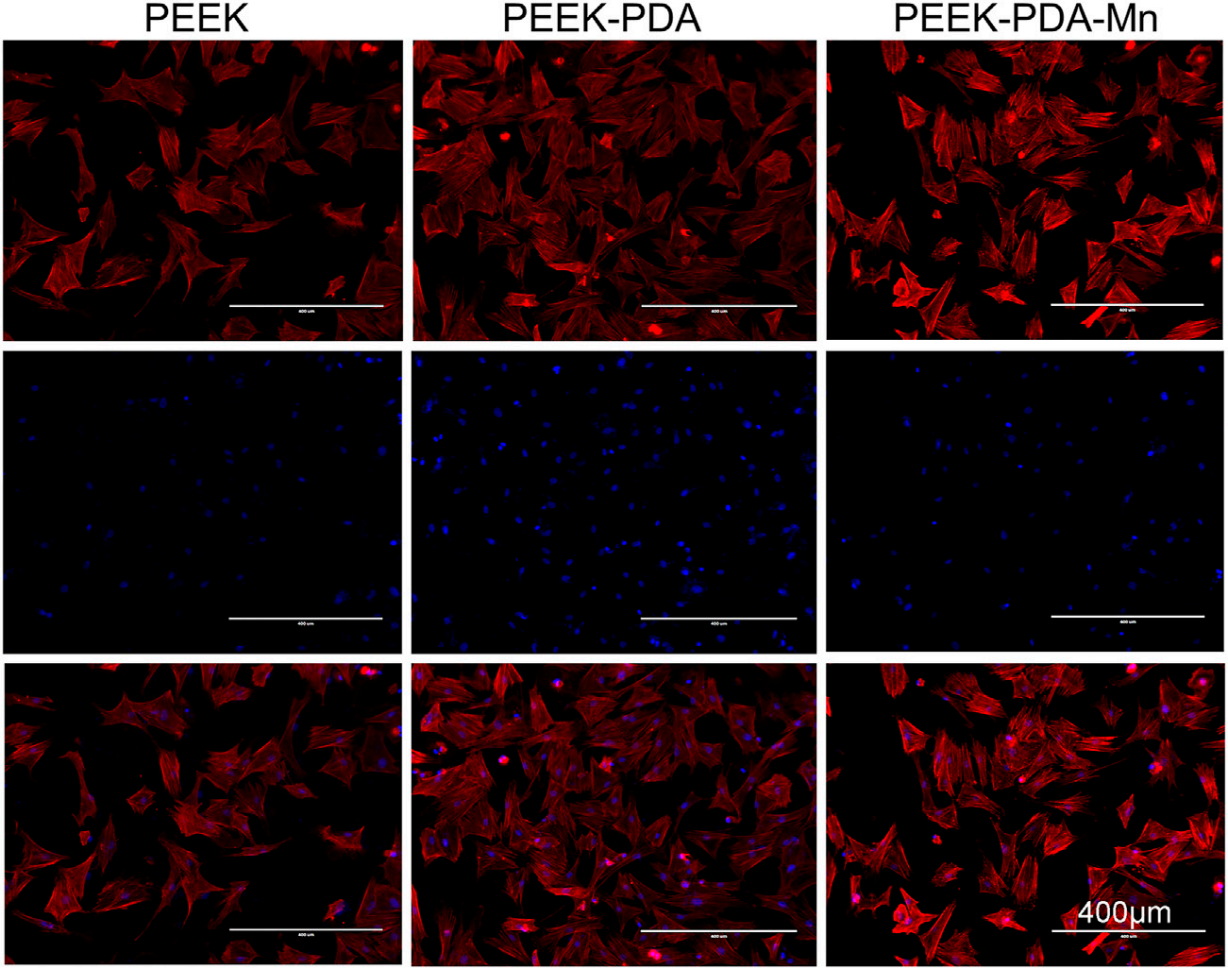
Fluorescent images of MC3T3-E1 cells cultured on different PEEKs for 24 h with actin stained with phalloidin (red) and nuclei stained with DAPI (blue), as observed using fluorescence microscope. Scale bars, 400 μm.

Fluorescence microscopy was utilized to view the cytoskeleton of MC3T3-E1 cells (Figure 5). PEEK-PDA-Mn and PEEK-PDA showed higher cell numbers on their surface than PEEK after culturing for 24 h, possibly as a result of the PDA coating’s enhanced biocompatibility. Additionally, MC3T3-E1 cells on PEEK group did not fully spread, and most of them were round or oval. There was a limited expression of F-actin in MC3T3-E1 cells. However, the cells on PEEK-PDA and PEEK-PDA-Mn surface spread sufficiently with a certain spindle shape, and a large number of lamellipodia and filopodia processes, indicating that PDA coating could stimulate cell attachment and spread. Additionally, there was no significant difference in cell morphology between PEEK-PDA and PEEK-PDA-Mn surface, which was consistent with the SEM results.

### 3.4 Cell viability

**FIGURE 6:**
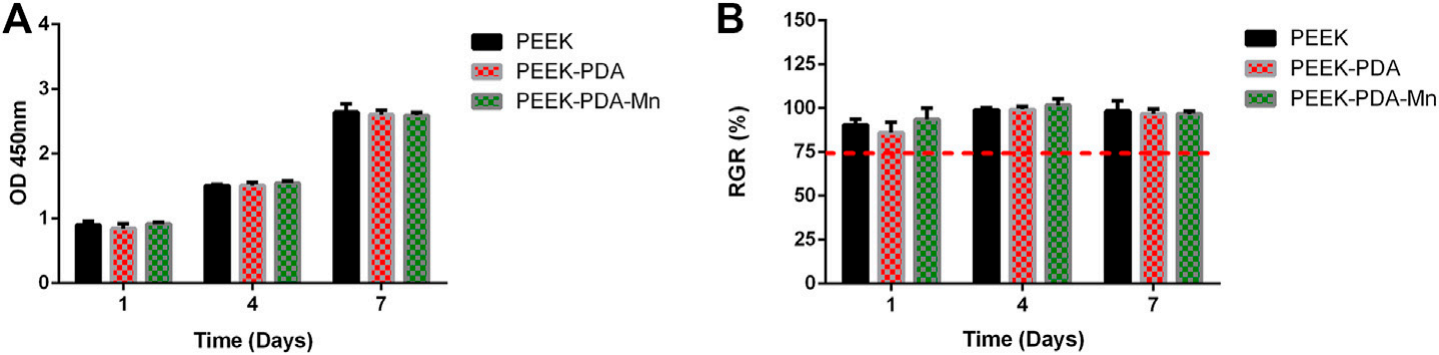
**(A)** OD values in CCK-8 tests of MC3T3-E1 cells cultured on PEEK, PEEK-PDA, and PEEK-PDA-Mn substrates for different times. **(B)** RGR values of MC3T3-E1 cells cultured on PEEK, PEEK-PDA, and PEEK-PDA-Mn substrates for different times.

The biocompatibility of the PEEKs was quantified by CCK-8 assay (Figure 6A). MC3T3-E1 cells on various PEEKs surfaces could proliferate stably without significant difference (*p* > 0.05) after culturing for 1, 4, and 7 days. As illustrated in Figure 6B, the relative growth rate of MC3T3-E1 cells on various PEEKs surface were higher than 75% at each time point, indicating that excellent biocompatibility of different modified PEEKs, which was consistent with SEM images and cytoskeletal staining results.

### 3.5 Osteogenesis evaluation

**FIGURE 7:**
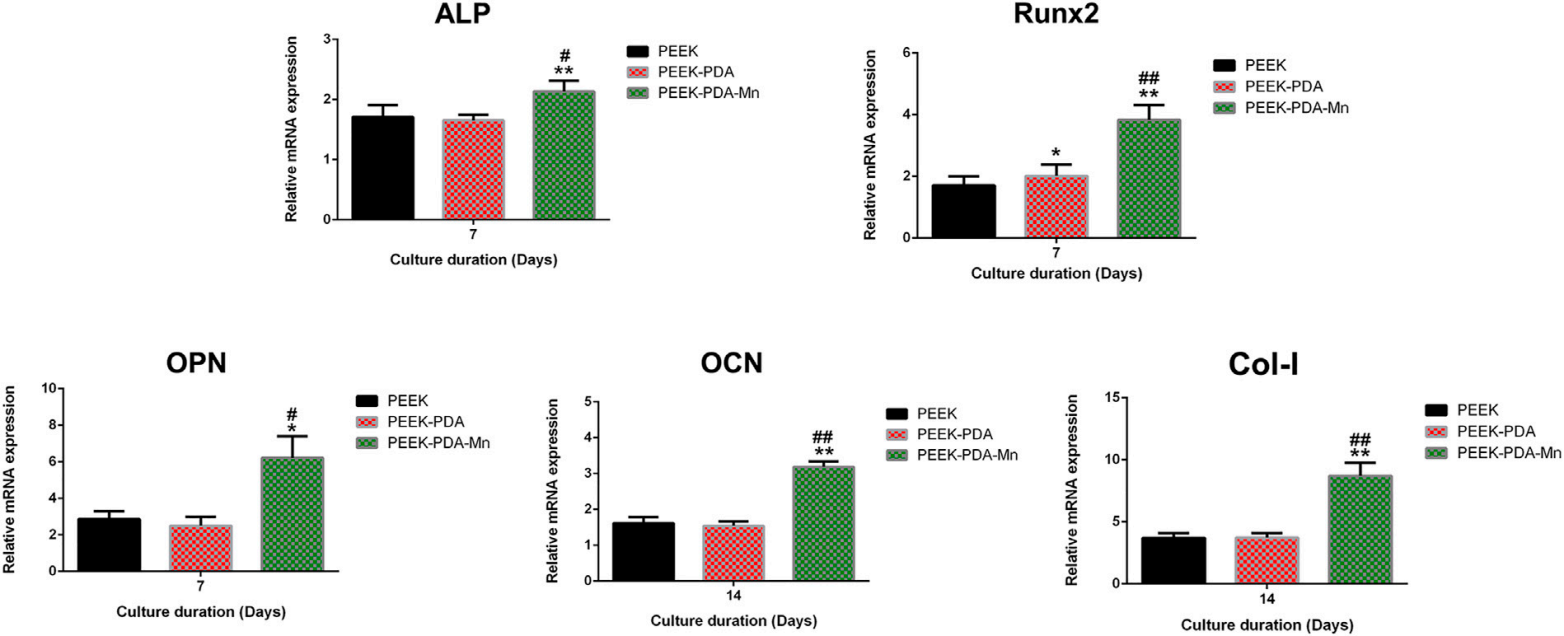
Osteogenic genes expression of MC3T3-E1 cells on various surfaces detected by RT-PCR at 7 and 14 days. **p < 0.01 and *p < 0.05 vs. PEEK; ##*p* < 0.01 and #*p* < 0.05 vs. PEEK-PDA.

To evaluate the osteogenic activities of PEEK with different modifications, RT-PCR was used to evaluate the expression level of osteogenic genes, including ALP, Runx2, OPN, OCN, and Col-I (Figure 7). After treatment of PEEK-PDA-Mn for 7 days, MC3T3-E1 cells showed upregulated gene expression in OPN, Runx2, and ALP. Moreover, the gene expression level of OCN and Col-I increased after culturing for 14 days. However, MC3T3-E1 cells cultured on PEEK without the modification of ions showed unchanged expression levels in the five osteogenesis-related genes.

**FIGURE 8:**
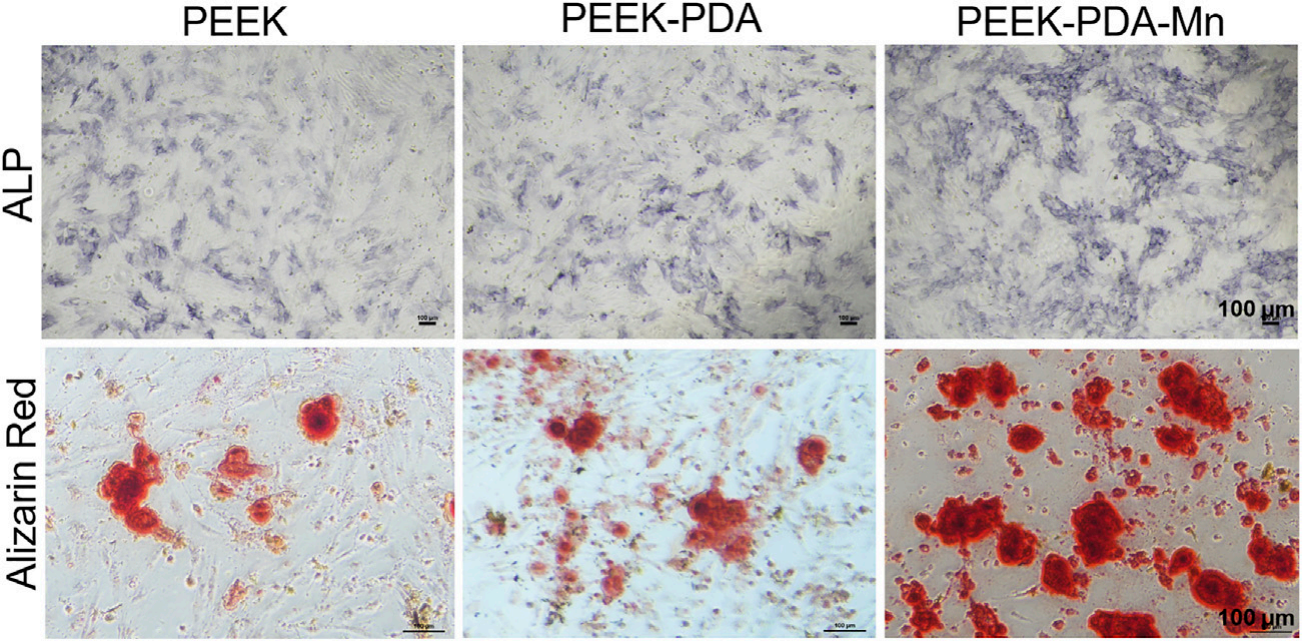
ALP staining and Alizarin Red staining of mineralized-ECM nodules produced by MC3T3-E1 cells at 7 and 14 days, respectively. Scale bars, 100 μm.

In this study, MC3T3-E1 cells were cultured for 7 and 14 days on different PEEKs, then ALP and alizarin red staining were utilized to evaluate the potential osteoinductive ability of PEEK with various modifications (Figure 8). After 7 days, the cells incubated on PEEK-PDA-Mn showed the highest ALP activity. After 14 days, the cells on PEEK and PEEK-PDA groups exhibited a slightly intense alizarin red staining, while a clearly intense stain was displayed on the PEEK-PDA-Mn group, suggesting the excellent osteogenesis induction of PEEK-PDA-Mn. Moreover, these staining results were supported by the RT-PCR results.

### 3.6 Bone regeneration *in vivo*

**FIGURE 9:**
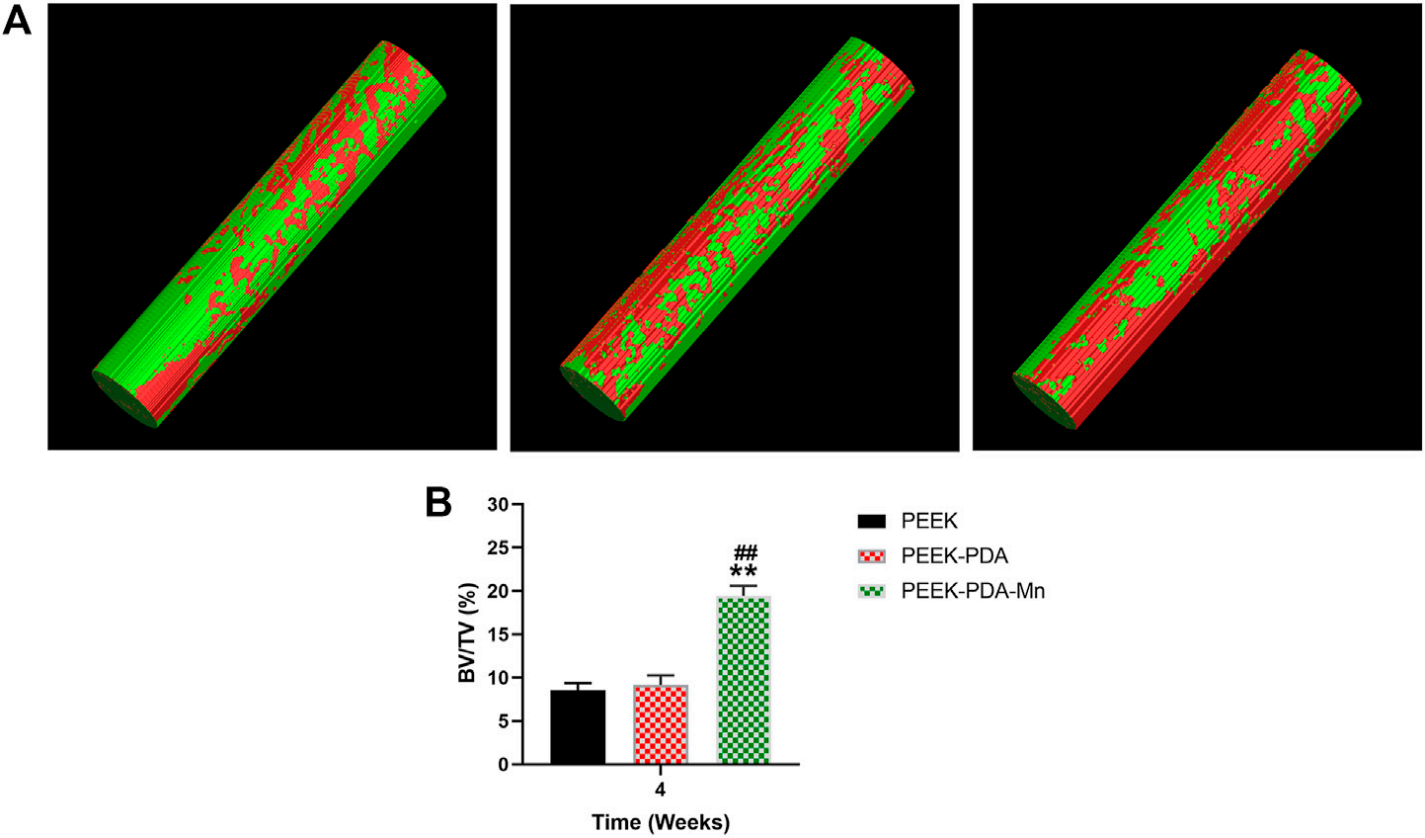
**(A)** Reconstructed Micro-CT images of new bone formation represented around various PEEK implants at 4 weeks after surgery. **(B)** Bone volume fraction (BV/TV) of the three groups obtained from analysis of the Micro-CT data (*n* = 4). **p < 0.01 vs. PEEK and ##*p* < 0.01 vs. PEEK-PDA.

The new bone formation ability of various PEEKs was evaluated 4 weeks after surgery. Figure 9A displays the reconstruction images of the femoral condyle defects implanted with the samples. The images indicated that small amounts of newly generated bones were found at the implant interfaces of PEEK-PDA and PEEK. In contrast, the highest amount of newly generated bones appeared at the interface of PEEK-PDA-Mn group. Figure 9B shows the quantification of bone formation of the samples. PEEK and PEEK-PDA exhibited the lowest bone volume fraction (BV/TV), PEEK-PDA-Mn exhibited the highest BV/TV. These results were ascribed to the osteogenic activities of manganese ions, and supported by the *in vitro* experiments.

**FIGURE 10:**
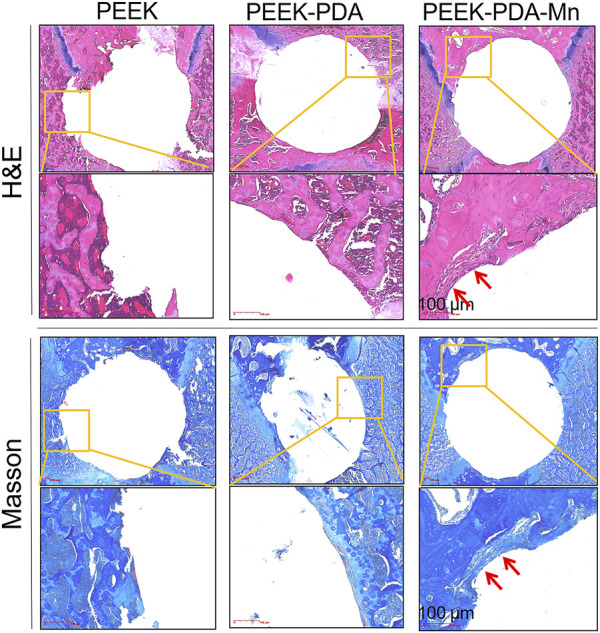
H&E and Masson’s trichrome staining of tissues surrounding various implants at 4 weeks post implantation. Scale bars, 100 μm.

After implantation with various PEEK implants, the H&E and Masson staining were utilized to evaluate newly formed bone and collagen tissue in the rat femur (Figure 10). Four weeks after surgery, H&E staining displayed that abundant newly generated bones with decreased fibrous tissue appeared on the interface between the peripheral tissue and PEEK-PDA-Mn materials (red arrow). In contrast, limited newly generated bones can be seen in the interface between PEEK and PEEK-PDA groups. Additionally, Masson staining exhibited that the interface between the implanted material and peripheral organization in PEEK and PEEK-PDA groups was full of a small amount of collagen tissue. However, the largest collagen tissue was seen in the PEEK-PDA-Mn group (red arrow), which was in accordance with the micro-CT results. These results indicated that PEEK-PDA-Mn possessed satisfactory properties in promoting bone regeneration *in vivo*.

## 4 Discussion

Due to various orthopedic diseases including trauma, infection, degeneration, tumor, etc. and their complications, bone defects are still major clinical challenges (Wang et al., 2018; Qi et al., 2021). There is an increasing demand for functional bone graft techniques worldwide. About $45 billion is annually spent on treating patients with bone-related diseases, which includes 1.6 million trauma-caused fractures and two million osteoporosis-related defects (O’Keefe and Mao, 2011). About 1.6 million patients receive bone transplantation repair surgery in the United States every year, costing about 2.5 billion dollars (Bharadwaz and Jayasuriya, 2020). The high rate of complications and reoperations, combined with functional deficits that limit these injuries, has profound clinical and economic implications (Nauth et al., 2018). Although bone tissue has the inherent potential of morphological and functional regeneration after injury, it will be disturbed by many pathological conditions. Currently, autologous bone, allograft bone, and xenografts are the main treatments for bone defects; each has benefits and drawbacks of its own. Autogenous bone has traditionally been regarded as the “gold standard,” which needs to collect bone from the patient and grafting to the damaged area of the same patient for bone repair (Garcia-Gareta et al., 2015). Nevertheless, autologous bone transplantation has many shortcomings, including limited available amount for transplantation, chronic pain, high incidence rate of donor area, secondary injury and infection, which affect the surgical effect (Du et al., 2018). Compared with autologous bone transplantation, the bone supply of allograft bone transplantation comes from the donor, which can be made into various sizes according to the clinical use needs, and there is no incidence rate of donor site (Noori et al., 2017). However, the risk of transmission of various infections is high and there is a possible immune response. Except for this, biologically inert materials like alumina, stainless steel, titanium alloys and polymethyl methacrylate, etc. are also employed for bone repair surgery (Noori et al., 2017; Gu et al., 2022). However, these materials may be encapsulated by fibrous tissue after implantation and fail to form integration with the host bone (Noori et al., 2017). In addition, the “stress shielding” effect caused by mismatched stiffness among the host bone and the implanted material leads to local osteoclast production and implant loosening, which limits its clinical use (Qi et al., 2021; Wang et al., 2022). Bone defect repair is a serious problem, which needs specific and expensive management. Therefore, the limitations of the current clinical treatment of bone defects require new treatment strategies with the aim of building biomaterials for repairing damaged bones, so as to reduce the complexity of the surgery, accelerate bone regeneration and improve the prognosis of patients.

Herein, the surface PEEK material containing manganese ions with excellent biocompatibility and promoting osteogenic activity was prepared by chemical modification of polydopamine. The biocompatibility and promoting osteogenic effect of PEEK materials were improved from both surface microstructure and local release induction. In terms of surface microstructure, hydrophilic surface and increased roughness of the polydopamine coating treatment can enhance biocompatibility of biomaterials, which is expected to promote cell adhesion and spreading. Previous studies reported that PDA coating improves the hydrophilicity and roughness of substrates and induces cell adhesion and growth (Jun et al., 2014; Huang et al., 2016; Ejeian et al., 2020). Regarding material composition, manganese ions with osteogenic activity were added on PEEK material surface, and the differentiation and proliferation of osteoblasts were further promoted through the release of manganese ions in the local microenvironment. EDS results confirm that Mn ions are anchored on the PDA coating through coordination and it is observed that manganese ions are continuously released from PEEK-PDA-Mn for 21 days by ICP-AES (Figures 2C, 3C). In this situation, the PDA coating avoids the explosive release of manganese ions, and can achieve its function of promoting bone tissue repair for a long time.

Evaluating the biocompatibility of coatings is as critical as evaluating the coatings’ potential in biomedical applications. Manganese is a crucial nutrient for intracellular activities and the average bone content was 1.7–3 PPM (Chen et al., 2018; Szurkowska et al., 2019). The beneficial mechanisms of manganese ions on bone metabolism include stimulation of osteocalcin, increase of ALP activity and production of Col-I (Huang et al., 2013; Szurkowska et al., 2019). Manganese has been proposed to play an important part in maintaining normal bone mass, and inhibiting bone loss in ovariectomized (Torres et al., 2014), which indicates its lack as a possible cause of osteoporosis (Bae and Kim, 2008; Szurkowska et al., 2019). However, in the study of osteoblasts *in vitro*, when the manganese content is 1wt%, the cell viability can be sharply reduced (Paluszkiewicz et al., 2010). In this study, we assessed the biocompatibility *in vitro* of various PEEKs utilizing MC3T3-E1 cells. It is crucial for cells to connect to the implant surface at early stage since it will influence later proliferation and differentiation of cells. The scanning electron microscopy and cytoskeletal observation results exhibited significant differences in the number as well as skeletal structure of MC3T3-E1 cells on various PEEKs surfaces (Figures 4, 5). The cell number on PEEK-PAD-Mn and PEEK-PDA surface were markedly higher in comparison to PEEK. Moreover, cell adhesion and spreading were improved in PEEK-PAD-Mn and PEEK-PDA cultured MC3T3-E1 cells compared to PEEK group, as evidenced by increased cell amount and irregular cell morphology, as well as the large number of lamellipodia and filopodia processes. These were ascribed to the improved surface characteristics including surface roughness and hydrophilicity as a result of PDA chemical modification (Huang et al., 2016; Ejeian et al., 2020). In addition, no significant difference in OD values was seen between various PEEKs, indicating the good biocompatibility of various PEEKs (Figure 6). Additionally, all of the PEEKs were well biocompatible with MC3T3-E1 cells at all culture times, as shown by RGR values that were higher than 75%. The Mn^2+^ exhibit concentration-dependent toxic effects on cells. For example, Luthen et al. discovered that ALP and Col-I mRNA expression levels were decreased and cytotoxicity was seen at high concentrations of manganese ions (>0.1 mM) (Luthen et al., 2007). Wang et al. (2021) reported that MC3T3-E1 cells did not exhibit any obvious cytotoxicity when exposed to Mn^2+^ (up to 50 g/mL) released from Mn-decorated biomaterials. In our study, ICP-AES was utilized to measure the release of Mn^2+^, and the maximum amount of Mn^2+^ measured at 21 days was 39.61 ± 3.31 μg/mL, which is consistent with the above research results. Therefore, the results above offered a compelling basis to support the good biocompatibility of PEEK-PDA-Mn coatings.

The ideal orthopedic implant requires osteogenic activity to allow bone tissue to grow in to stabilize the prosthesis. Without osteogenic activity, implants may face poor deposition of new bone tissue and subsequent aseptic loosening (Raphel et al., 2016). To determine the molecular mechanisms that promote osteogenic differentiation of MC3T3-E1 cells cultured on various PEEKs, the expression of osteogenic genes was investigated through RT-PCR. The expression levels of osteogenic genes in PEEK-PDA-Mn group were higher (Figure 7) in contrast to PEEK and PEEK-PDA groups. It is crucial for orthopedic implant to have the ability to induce cells to mineralize matrix and nodules. Thus, we evaluated the mineralization on MC3T3-E1 cells that incubated with various PEEK by ALP and alizarin red staining (Figure 8). After 7 days, PEEK-PDA-Mn exhibited higher ALP activity compared to that of PEEK-PDA and PEEK. Furthermore, after 14 days, MC3T3-E1 cells cultivated with PEEK-PDA-Mn exhibited more calcium nodules in comparison to PEEK-PDA and PEEK. The alizarin red and ALP staining results were consistent with the PCR results. The upregulated expression of ALP gene is essential to phosphate formation, which helps to bone formation (Zhu et al., 2019). This may explain the results that MC3T3-E1 cells cultured on PEEK-PDA-Mn showed the highest expression level of ALP. In addition, enhanced expression of OCN gene, which serves as a later marker of osteoblast differentiation, suggests that PEEK-PDA-Mn promotes and maintains bone nodules formation (Yu et al., 2020). This could answer why there are more calcium nodules on PEEK-PDA-Mn surface. This was also supported by a considerable number of earlier investigations have demonstrated that Mn^2+^ as an additive can be used to modify various biomaterials to produce biological activity of promoting osteogenic (Barrioni et al., 2019; Zhao et al., 2020; Wang et al., 2021). Therefore, these results indicate that PEEK-PDA-Mn is superior to PEEK and PEEK-PDA in promoting osteogenesis.

New orthopedic implants require efficient *in vivo* evaluation tools and methods for research and development. Animal models can simulate microenvironment of bone growth, and have significant advantages in assessing the osteogenic properties of materials (Lin et al., 2017). Moreover, animal models are an indispensable bridge between laboratory and clinical trials. Osseointegration was defined as the direct functional and structural connection between the inner plant surface and bone tissue (Albrektsson and Albrektsson, 1987). An effective osseointegration is crucial to the long-term maintenance of prosthesis. Here, we chose the intramedullary implantation method to assess the bone integration ability of the implant *in vivo*. At present, most animal studies begin to observe the osteogenesis of the implant *in vivo* 1 month after surgery (Lin et al., 2017). In comparison to X-ray photography, micro-CT analysis provides a quantitative assessment of bone formation in three dimensions and may acquire a depth picture of bone growth regions to track the evolution of osteogenesis. 3D reconstruction of scan data through imaging processing and quantitative measurement and analysis of ROI (such as volume, relative density, etc.) software allows researchers to accurately compare bone growth. Therefore, despite the high cost, Micro-CT has been widely employed in bone tissue-related studies (Odekerken et al., 2013; Lin et al., 2017). Figure 9 showed the Micro-CT results of newly formed bone. The results showed that the BV/TV of PEEK-PDA-Mn was significantly higher in comparison to PEEK and PEEK-PDA groups after surgery for 4 weeks, which was consistent with the results that Mn-containing biomaterials promote osteogenesis *in vivo* (Barrioni et al., 2019; Zhao et al., 2020; Wang et al., 2021). Because it offers clear and comprehensive data of osteogenesis, histological analysis is the most common method of assessment. At present, Masson and H&E staining are widely used to study osteogenesis (Odekerken et al., 2013; Lin et al., 2017). After implantation for 4 weeks, the interface among the implanted material and peripheral organization in PEEK and PEEK-PDA groups was filled by a modest quantity of newly produced bone and collagen tissue, according to histological analysis using Masson and H&E staining. However, the PEEK-PDA-Mn group showed the biggest amounts of newly formed mature bone and collagen (Figure 10). Taken together, the animal model of bone defect with implant integration suggested that the PEEK-PDA-Mn possessed an excellent capacity to stimulate osseointegration *in vivo*.

The results of the experiments *in vitro* and *in vivo* showed that PEEK-PDA-Mn has good biocompatibility and enhanced bone integration. For PEEK to be used in orthopedic and tissue engineering scaffolds going forward, this study offers an experimental and theoretical foundation. Additionally, the universality of the mussel adhesion process that is mirrored in our approach might offer a broad surface bioengineering technique for a wider variety of biomedical implants.

## 5 Conclusion

In this study, a PDA adhesion coating method was used to successfully immobilize manganese on the PEEK surface via a PDA adhesion coating approach. By adding PDA and manganese, the surface characteristics (e.g., roughness and hydrophilicity) and biocompatibility of the PEEK-PDA-Mn groups were markedly improved. According to the results of osteogenic genes expression, ALP and mineralization indicate that manganese immobilization can considerably enhance MC3T3-E1 cells’ ability to differentiate into osteoblasts *in vitro*. The ability of the various PEEK implants to promote *in vivo* new bone growth was assessed using a rat model of femoral condyle bone deficiency. The Micro-CT and histological analysis results suggested that the PEEK-PDA-Mn implants exhibited a promising ability in promoting bone tissue regeneration in defect area.

## Data Availability

The original contributions presented in the study are included in the article/supplementary material, further inquiries can be directed to the corresponding authors.
