# Corrigendum: Coating of manganese functional polyetheretherketone implants for osseous interface integration

**DOI:** 10.3389/fbioe.2023.1232967

**Published:** 2023-06-20

**Authors:** Xin Yang, Shouliang Xiong, Jing Zhou, Yinchang Zhang, Huazheng He, Pingbo Chen, Congming Li, Qiang Wang, Zhiqiang Shao, Lei Wang

**Affiliations:** ^1^ Department of Orthopedics, The First Affiliated Hospital of Wannan Medical College, Wuhu, Anhui, China; ^2^ Orthopedics and Sports Medicine Center, The Affiliated Suzhou Hospital of Nanjing Medical University, Suzhou, China

**Keywords:** PEEK, manganese, polydopamine, biocompatibility, osseointegration

In the published article, there was an error in Figure 4 as published. To reveal the cell morphology of MC3T3-E1 on the substrate of PEEK-PDA-Mn, it had been accidentally replaced by the acquired image of the PEEK-PDA-Mn group when we used AI software to typeset for manuscript preparing. The corrected Figure 4 and its caption appear below.

At the same time, the misused image is also used as part of the schematic diagram of the article. Therefore, corresponding correction have been made to the schematic diagram of Figure 1. The corrected Figure 1 and its caption are shown as below.

**FIGURE 1:**
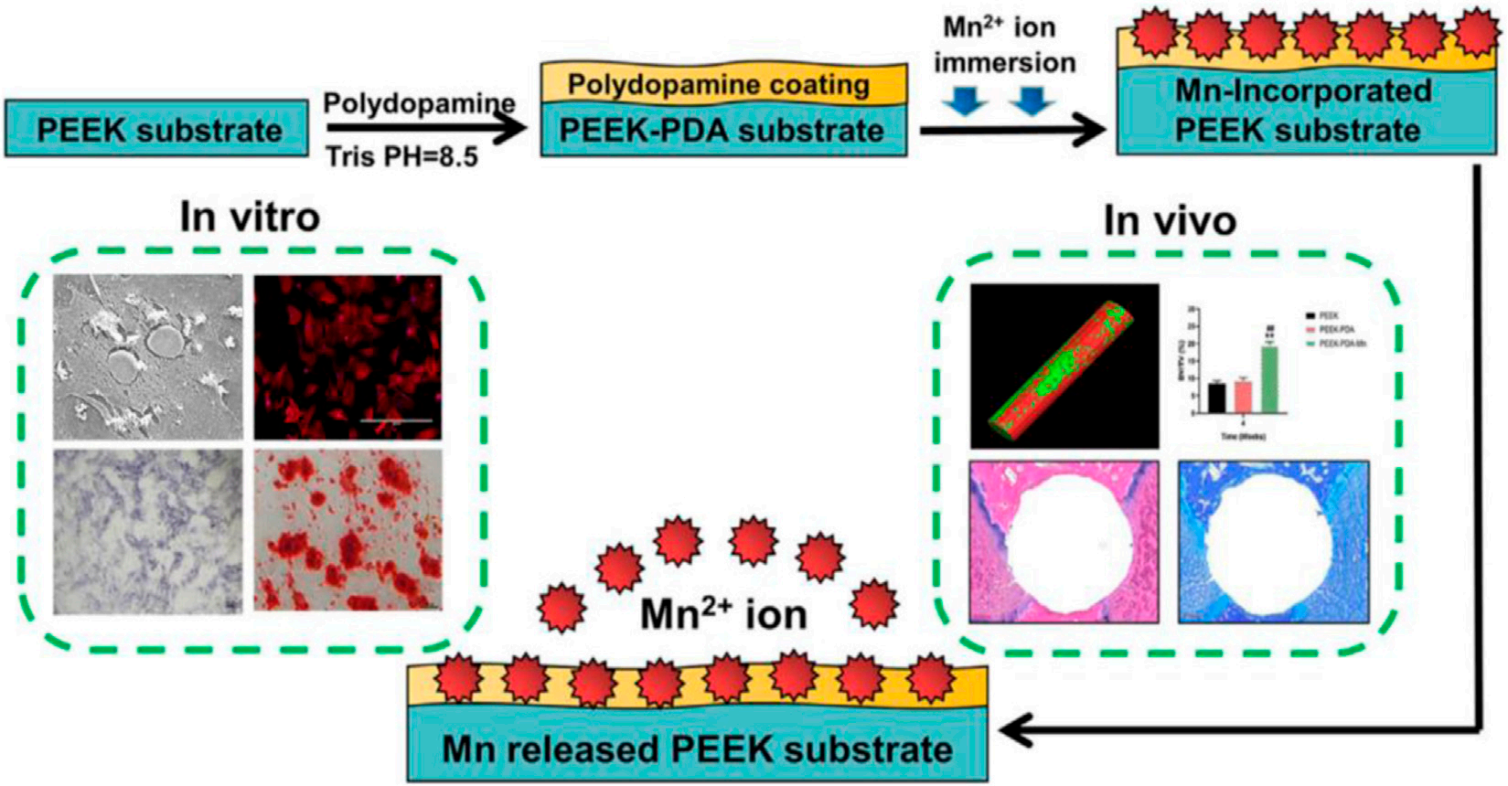
Schematic diagram of the process used for preparation and evaluation of the PEEK-PDA-Mn materials.

**FIGURE 4:**
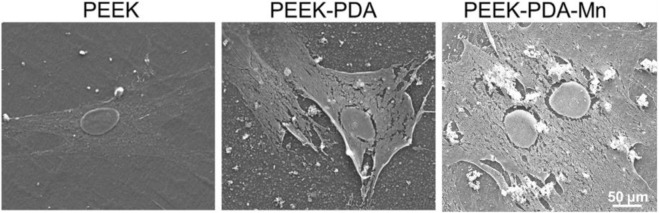
Cell morphology ofMC3T3-E1 cells cultured on PEEK, PEEK-PDA, and PEEK-PDA-Mn substrates for 24 h, as observed using SEM. Scale bars, 50 μm.

The authors apologize for this error and state that this does not change the scientific conclusions of the article in any way. The original article has been updated.

